# Metabolic engineering of *Saccharomyces cerevisiae* for production of fatty acid short- and branched-chain alkyl esters biodiesel

**DOI:** 10.1186/s13068-015-0361-5

**Published:** 2015-11-04

**Authors:** Wei Suong Teo, Hua Ling, Ai-Qun Yu, Matthew Wook Chang

**Affiliations:** Department of Biochemistry, Yong Loo Lin School of Medicine, National University of Singapore, 14 Medical Drive, Singapore, 117597 Singapore; NUS Synthetic Biology for Clinical and Technological Innovation (SynCTI), Life Sciences Institute, National University of Singapore, 28 Medical Drive, Singapore, 117456 Singapore

**Keywords:** Metabolic engineering, Synthetic biology, Yeast, Biofuel, Biodiesel, Fatty acid short-chain alkyl esters, Fatty acid branched-chain alkyl esters

## Abstract

**Background:**

Biodiesel is a mixture of fatty acid short-chain alkyl esters of different fatty acid carbon chain lengths. However, while fatty acid methyl or ethyl esters are useful biodiesel produced commercially, fatty acid esters with branched-chain alcohol moieties have superior fuel properties. Crucially, this includes improved cold flow characteristics, as one of the major problems associated with biodiesel use is poor low-temperature flow properties. Hence, microbial production as a renewable, nontoxic and scalable method to produce fatty acid esters with branched-chain alcohol moieties from biomass is critical.

**Results:**

We engineered *Saccharomyces cerevisiae* to produce fatty acid short- and branched-chain alkyl esters, including ethyl, isobutyl, isoamyl and active amyl esters using endogenously synthesized fatty acids and alcohols. Two wax ester synthase genes (*ws2* and *Maqu_0168* from *Marinobacter sp.*) were cloned and expressed. Both enzymes were found to catalyze the formation of fatty acid esters, with different alcohol preferences. To boost the ability of *S. cerevisiae* to produce the aforementioned esters, negative regulators of the *INO1* gene in phospholipid metabolism, Rpd3 and Opi1, were deleted to increase flux towards fatty acyl-CoAs. In addition, five isobutanol pathway enzymes (Ilv2, Ilv5, Ilv3, Aro10, and Adh7) targeted into the mitochondria were overexpressed to enhance production of alcohol precursors. By combining these engineering strategies with high-cell-density fermentation, over 230 mg/L fatty acid short- and branched-chain alkyl esters were produced, which is the highest titer reported in yeast to date.

**Conclusions:**

In this work, we engineered the metabolism of *S. cerevisiae* to produce biodiesels in the form of fatty acid short- and branched-chain alkyl esters, including ethyl, isobutyl, isoamyl and active amyl esters. To our knowledge, this is the first report of the production of fatty acid isobutyl and active amyl esters in *S. cerevisiae*. Our findings will be useful for engineering *S. cerevisiae* strains toward high-level and sustainable biodiesel production.

**Electronic supplementary material:**

The online version of this article (doi:10.1186/s13068-015-0361-5) contains supplementary material, which is available to authorized users.

## Background

Biodiesel is a mixture of fatty acid short-chain alkyl esters of different fatty acid carbon chain lengths. FAMEs (fatty acid methyl esters) and FAEEs (fatty acid ethyl esters) are used as commercial biodiesel and obtained via transesterification of vegetable oils with an alcohol (methanol or ethanol) with the aid of a catalyst. However, while FAMEs or FAEEs are useful alternative diesel fuels currently being used in the market, fatty acid esters with branched-chain alcohol moieties have better fuel properties [1, 2]. Crucially, this includes improved cold flow characteristics where cloud points and pour points are reduced, as one of the major problems associated with biodiesel use is poor low-temperature flow properties [3].

Baker’s yeast *Saccharomyces cerevisiae*, which is used for industrial scale bioethanol production, brewing and winemaking, is an important host for biotechnological applications [4]. The ability to grow robustly and the abundance of genetic tools available for its manipulation make yeast an ideal host microbe for engineering biofuel production. Various research groups have engineered yeast for production of FAEEs by heterologous expression of an acyl-CoA: alcohol acyltransferase or wax ester synthase [5–11]. Five wax ester synthases, from *Acinetobacter baylyi* ADP1*, Marinobacter hydrocarbonoclasticus* DSM 8798*, Rhodococcus opacus* PD630*, Mus musculus* C57BL/6 *and Psychrobacter arcticus* 273-4 were expressed in yeast where wax ester synthase from *M. hydrocarbonoclasticus* DSM 8798 (ws2) was found to produce the most FAEEs (6.3 mg/L) [5]. Multiple metabolic engineering strategies were applied towards boosting the production of FAEEs in yeast. Abolishing protein kinase Snf1-dependent regulation of acetyl-CoA carboxylase Acc1 through Ser659 and Ser1157 mutation increased FAEEs titers to 15.8 mg/L [6]. Elimination of non-essential fatty acid utilization pathways through deletion of acyl-CoA: sterol acyltransferases (encoded by *ARE1* and *ARE2*), diacylglycerol acyltransferases (encoded by *DGA1* and *LRO1*) and fatty acyl-CoA oxidase (encoded by *POX1*) led to 17.2 mg/L FAEEs [7]. Integration of 6 copies of the ws2 expression cassette into the genome increased production of FAEEs to 34 mg/L, while further boosting of the fatty acyl-CoAs availability by acyl-CoA binding protein (encoded by *ACB1*) overexpression and NADPH supply by overexpression of bacterial NADP^+^-dependent glyceraldehyde-3-phosphate dehydrogenase (*gapN*) led to FAEEs production of 48 mg/L [8].

**Fig. 1:**
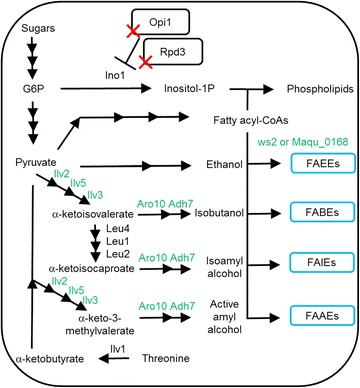
Metabolic engineering strategy to produce FASBEs. FASBEs can be produced by expressing a wax ester synthase (ws2 or Maqu_0168). Isobutanol pathway enzymes (Ilv2, Ilv5, Ilv3, Aro10, Adh7) were overexpressed in the mitochondria to accumulate more isobutanol and FABEs. At the same time, production of isoamyl alcohol and active amyl alcohols were also increased, resulting in increased FAIEs and FAAEs production. To boost FASBEs production, negative regulators of *INO1* (Opi1, Rpd3) were deleted. Genes overexpressed are shown in *green*. *Red crosses* gene deletions

On the other hand, only trace amounts of fatty acid isoamyl esters (FAIEs) and FAEEs were produced in an engineered yeast strain expressing wax ester synthase from *A. baylyi ADP1* and with *ARE1*, *ARE2*, *DGA1* and *LRO1* disrupted [12, 13]. In addition, the metabolic engineering of yeast to produce and accumulate fatty acid isobutyl esters (FABEs) and fatty acid active amyl esters (FAAEs) has not been reported. Here, we engineered yeast to produce fatty acid short- and branched-chain esters (FASBEs), including ethyl, isobutyl, active amyl and isoamyl esters, using endogenously synthesized fatty acids and alcohols (Fig. 1). First, two wax ester synthase genes (*ws2* and *Maqu_0168* from *Marinobacter sp.*) were cloned and expressed. Second, negative regulators of the *INO1* gene in phospholipid metabolism, Rpd3 and Opi1, were deleted. *INO1* gene encodes for inositol-3-phosphate synthase that makes inositol phosphates and inositol-containing phospholipids. As synthesis of phospholipids requires fatty acyl-CoAs as precursors, the removal of *INO1* negative regulators may boost flux towards fatty acyl-CoAs-derived phospholipids and the abovementioned esters [14].The deletion of *RPD3* and *OPI1* was shown previously to enable simultaneous increase of phospholipids and desired product 1-hexadecanol [15]. Third, isobutanol pathway enzymes (acetolactate synthase Ilv2, ketoacid reductoisomerase Ilv5, dihydroxyacid dehydratase Ilv3, α-ketoacid decarboxylase Aro10, and alcohol dehydrogenase Adh7) targeted into the mitochondria were overexpressed to boost production of alcohol precursors. Ilv2, Ilv5 and Ilv3 are naturally located in the mitochondria, whereas Aro10 and Adh7 were re-targeted to the mitochondria using N-terminal fusion with mitochondria localization signal from subunit IV of the yeast cytochrome c oxidase (encoded by *COX4*) [16, 17]. Compartmentalization of this pathway into the mitochondria enabled high-level production of branched-chain alcohols. Finally, by combining these engineering strategies with high-cell-density fermentation, over 230 mg/L FASBEs were produced, which represents the highest titer reported in yeast to date.

## Results and discussion

### Substrate preference of the wax ester synthases

**Fig. 2:**
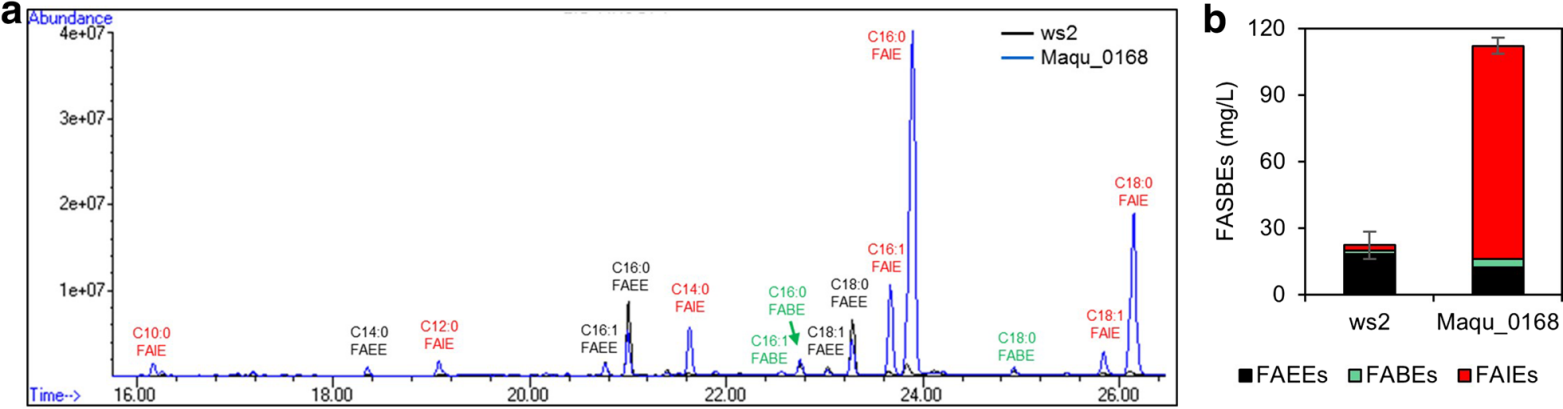
Substrate preference of the wax ester synthases. **a** Representative GC/MS result of FASBEs produced in BY4742 expressing *ws2* or *Maqu_0168*. **b** Corresponding FASBEs titers. Values are the mean of biological triplicates ±SD after 48 h

To produce FASBEs in *S. cerevisiae*, a wax ester synthase gene needs to be expressed. The ws2 enzyme, which was selected as the best wax ester synthase for FAEEs production in yeast [5], and the Maqu_0168 enzyme, which was shown in vitro to have high wax synthase activities [18], were, respectively, cloned into pESC-URA plasmid for expression in yeast. It was found that the yeast strain expressing *ws2* produced mostly FAEEs (18.2 mg/L), with a small proportion of FABEs (1.85 mg/L) and FAIEs (2.21 mg/L) produced (Fig. 2). On the other hand, the yeast strain expressing *Maqu_0168* resulted in a large proportion of FAIEs (96.2 mg/L), less FAEEs (12.2 mg/L), and a small amount of FABEs (3.82 mg/L). Further, as shown from the gas chromatography mass spectrometry (GC/MS) peaks (Fig. 2), the majority of the products were C16 and C18 fatty acid derived.

To determine whether having more alcohols present in the culture medium can further boost FASBEs product levels, 0.1 % ethanol, isobutanol or isoamyl alcohol was added exogenously. Additional file 1: Figure S1A shows that for the yeast strain expressing *ws2*, additional ethanol did not result in increased FAEEs titers, whereas additional isobutanol and isoamyl alcohol did result in ninefold increase in FABEs and FAIEs product titers, respectively. Therefore, while ethanol is not rate limiting in the yeast strains expressing *ws2* for FAEEs production, isobutanol and isoamyl alcohol levels are rate limiting for FABEs and FAIEs production. Additional file 1: Figure S1B shows that for the yeast strain expressing *Maqu_0168*, neither exogenous ethanol nor isoamyl alcohol resulted in increased FAEEs or FAIEs titers, respectively, whereas additional isobutanol increased FABEs levels by eightfold. Hence, both ethanol and isoamyl alcohol are not rate limiting in the yeast strain expressing *Maqu_0168*, but isobutanol is rate limiting for FABEs production. In addition, this result indicates that Maqu_0168 has higher affinity for isoamyl alcohol compared to ws2.

### Deletion of *INO1* negative regulators to boost FASBEs production

**Fig. 3:**
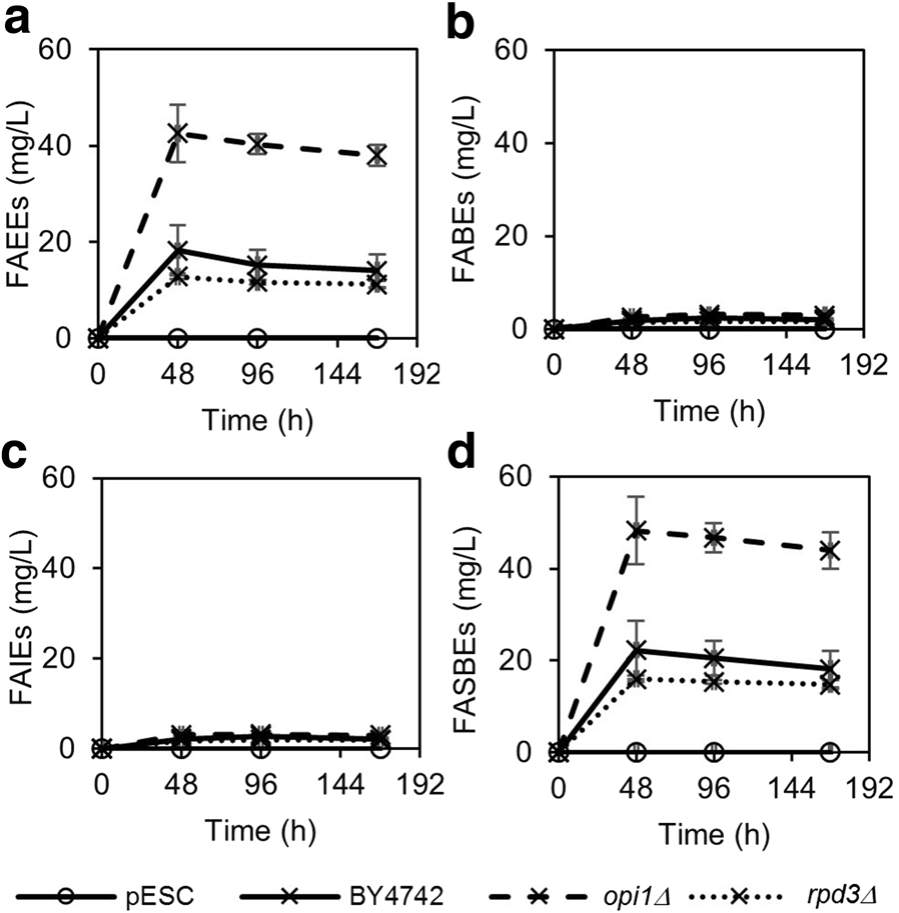
Production of **a** FAEEs, **b** FABEs, **c** FAIEs and **d** Total FASBEs, in BY4742 and knockout strains of *INO1* negative regulators (*rpd3Δ* and *opi1Δ*) expressing wax ester synthase ws2. All strains were cultured in minimal medium lacking uracil with 0.2 % glucose and 1.8 % galactose. Values are the mean of biological triplicates ±SD at 0, 48, 96, and 168 h. Figures with adjusted y-axis scales for (**b**) and (**c**) are shown in Additional file 1: Figure S2

Towards engineering yeast strains that produce high levels of fatty acid esters, we deleted two genes that are *INO1* negative regulators, namely *RPD3* and *OPI1*. Deletion of both genes was shown previously to boost phospholipid production in yeast, and simultaneously boost fatty acyl-CoA derived fatty alcohol titers [15]. Figure 3 shows the production of FAEEs, FABEs, FAIEs and total FASBEs in the yeast strains and mutants expressing *ws2* over a period of 168 h. Fermentation of yeast was followed up to 168 h to enable yeast cells to biosynthesize and accumulate more products. Here, we use values obtained at 168 h to discuss the production levels of FABSEs. While production of FABEs and FAIEs is low in the wild-type strain (2.0 mg/L for FABEs and 2.1 mg/L for FAIEs), FAEEs production was higher at 14.0 mg/L. When *OPI1* was deleted, the production of FAEEs increased 2.7-fold to 38.0 mg/L. However, deletion of *RPD3* reduced fatty acid esters production, as opposed to the case for fatty alcohol production [15]. By carrying out a phospholipid assay, we found that indeed under our test conditions, *OPI1* deletion increased phospholipid levels whereas *RPD3* deletion decreased phospholipid levels (Additional file 1: Figure S3). This is possibly due to a difference in growth conditions, where glucose was used as carbon source in the previous report, while here, we have used galactose as inducer of protein expression and carbon source for growth. In the *OPI1* knockout strain, a total FASBEs titer of 43.9 mg/L was obtained.

**Fig. 4:**
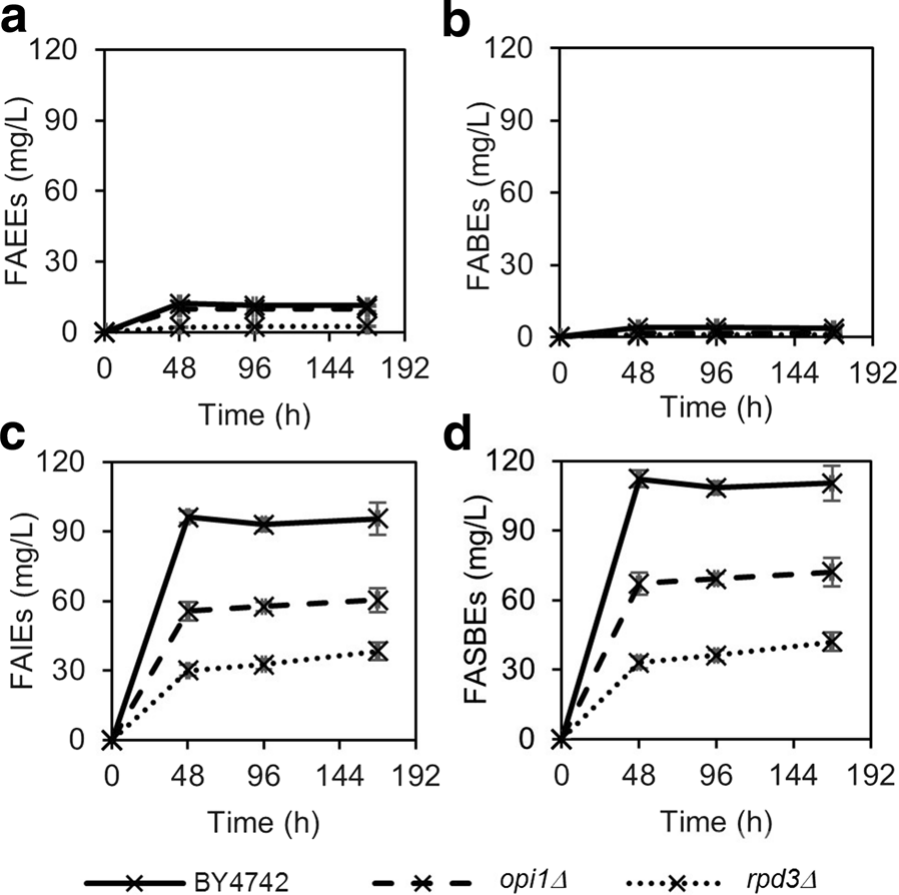
Production of **a** FAEEs, **b** FABEs, **c** FAIEs and **d** Total FASBEs, in BY4742 and knockout strains of *INO1* negative regulators (*rpd3Δ* and *opi1Δ*) expressing wax ester synthase Maqu_0168. All strains were cultured in minimal medium lacking uracil with 0.2 % glucose and 1.8 % galactose. Values are the mean of biological triplicates ±SD at 0, 48, 96, and 168 h. Figures with adjusted y-axis scales for (**a**) and (**b**) are shown in Additional file 1: Figure S4

Figure 4 shows the production of FAEEs, FABEs, FAIEs, and total FASBEs in the yeast strains and mutants expressing *Maqu_0168* over a period of 168 h. Here, the production of FAEEs and FABEs is low in the wild-type strain (11.3 mg/L for FAEEs and 3.4 mg/L for FABEs) compared to the production of FAIEs (95.6 mg/L). The total FASBEs produced was 110.3 mg/L. Also, it was found that deletion of *OPI1* and *RPD3* both resulted in reduced FASBEs production levels. As the production of fatty acid esters requires two substrates, fatty acyl-CoA and alcohol, a possible explanation is that deletion of *OPI1* caused a reduction in isoamyl alcohol levels. This was confirmed by quantifying alcohol levels, where BY4742 *opi1Δ* (45.0 mg/L) had lower isoamyl alcohol levels compared to BY4742 (64.3 mg/L) (Table 1).

**Table 1:** Alcohol production of engineered yeast strains. Values are the mean of biological triplicates ± standard deviation after 48 h

Alcohols	Strain				
	BY4742	BY4742 *opi1Δ*	BY4742 *rpd3Δ*	BY4742-IB	BY4742-IB *opi1Δ*
Ethanol (g/L)	3.43 ± 0.24	7.17 ± 0.17	7.42 ± 0.45	4.39 ± 0.29	4.36 ± 0.11
Isobutanol (mg/L)	9.61 ± 0.58	5.01 ± 0.62	11.6 ± 1.00	176.6 ± 9.54	178.1 ± 9.74
Isoamyl alcohol (mg/L)	64.3 ± 3.82	45.0 ± 2.20	68.6 ± 5.04	90.1 ± 2.91	87.2 ± 10.5
Active amyl alcohol (mg/L)	0.22 ± 0.05	0.13 ± 0.01	0.21 ± 0.01	25.2 ± 0.35	26.2 ± 3.66

Both Figs. 3 and 4 show that fatty acid ester titers were comparable at 48, 96 and 168 h. This suggests that production of FASBEs occurs mainly during cell growth stage, and is greatly reduced or stopped in stationary phase (Additional file 1: Figure S5). In addition, FAEEs titers were higher at 48 h and decreased after that, suggesting that FAEEs may have diffused out of the cell, or possibly degraded. However, extraction of products in the medium did not yield any FAEEs, indicating that most FAEEs were intracellular, while extracellular FAEEs had likely evaporated. Similarly, no FABEs or FAIEs were detected in the medium.

### Boosting FABEs production

As seen in Figs. 3 and 4, the production of FABEs is minimal. Hence, to boost FABEs production, genes that enable the production and accumulation of isobutanol need to be expressed in yeast. Here, we obtained a yeast strain (BY4742-IB) that has five isobutanol pathway enzymes (Ilv2, Ilv5, Ilv3, Aro10 and Adh7) overexpressed in the mitochondria of yeast to supply the required isobutanol [17]. This strain was constructed through integration of expression cassettes into δ-sites of retrotransposon elements of yeast chromosomes. Isobutanol produced is naturally transported out from the mitochondria into the cytoplasm where synthesis of FASBEs takes place. The increased isobutanol production was confirmed where an 18-fold increase to 176.6 mg/L isobutanol was obtained compared to BY4742 (9.61 mg/L) (Table 1).

**Fig. 5:**
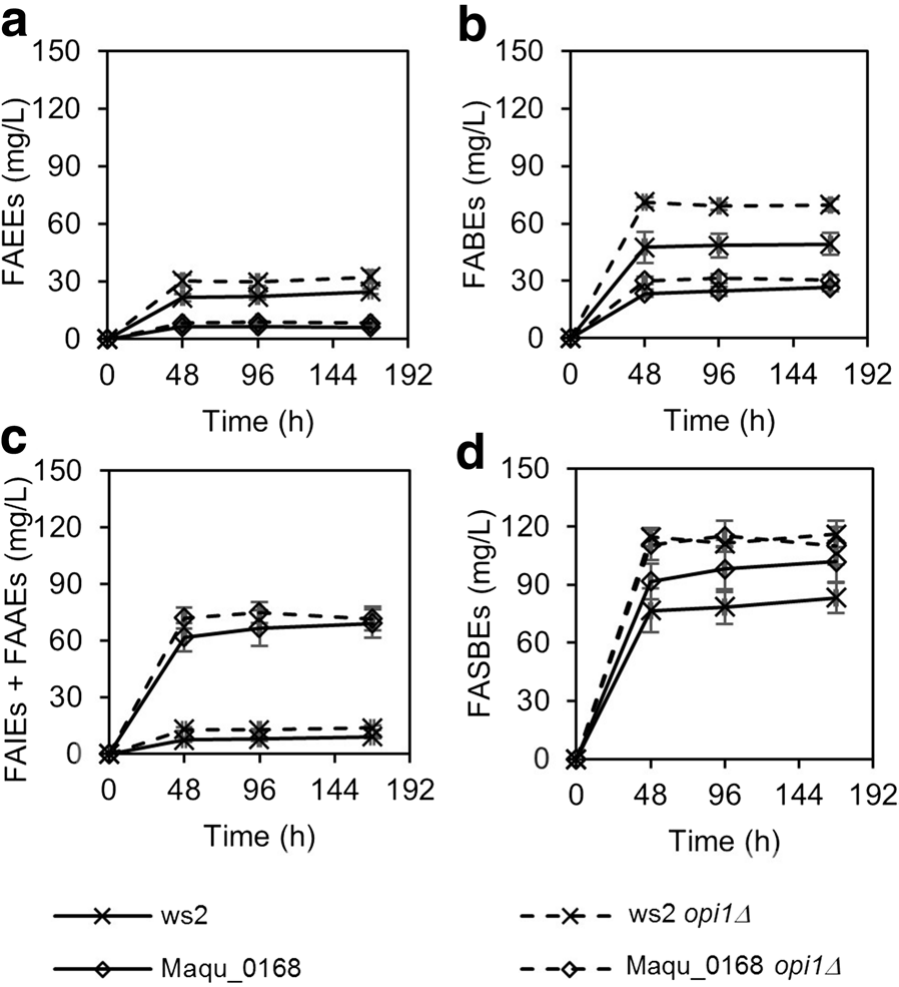
Production of **a** FAEEs, **b** FABEs, **c** FAIEs and FAAEs, and **d** Total FASBEs, in BY4742-IB and BY4742-IB *opi1Δ* expressing wax ester synthase ws2 or Maqu_0168. All strains were cultured in minimal medium lacking uracil and valine with 0.2 % glucose and 1.8 % galactose. Values are the mean of biological triplicates ±SD at 0, 48, 96, and 168 h

We then expressed ws2 and Maqu_0168 wax ester synthases in BY4742-IB and its *OPI1* knockout mutant (Fig. 5). A *RPD3* knockout mutant was not used because *RPD3* deletion did not increase FASBEs production in BY4742. BY4742-IB expressing *ws2* produced 49.3 mg/L FABEs, whereas BY4742-IB expressing *Maqu_0168* produced 26.6 mg/L FABEs. Also, the deletion of *OPI1* increased the amount of FABEs produced for both *ws2* and *Maqu_0168* expressing strains to 69.8 and 30.2 mg/L, respectively.

At the same time, in BY4742-IB strains, FAAEs were also accumulated when active amyl alcohols levels were produced (Table 1). The production of active amyl alcohol is a result of overlapping synthesis pathway with the isobutanol producing pathway [16]. FAAEs were not detected in wild-type BY4742 strain due to the absence of active amyl alcohol. In BY4742-IB *opi1Δ* expressing *ws2*, 4.25 mg/L FAAEs were produced, whereas 3.01 mg/L FAAEs were obtained when *Maqu_0168* was expressed. FAIEs were the major fatty acid amyl esters produced, where production levels of FAIE for BY4742-IB *opi1Δ* expressing *ws2* and *Maqu_0168* were 9.6 mg/L and 68.7 mg/L, respectively. For FAEEs, 32.4 mg/L was produced in BY4742-IB *opi1Δ* expressing *ws2*, whereas 8.3 mg/L was produced in BY4742-IB *opi1Δ* expressing *Maqu_0168*. The total FASBEs produced increased for both *ws2* (83.2 mg/L to 116.1 mg/L) and *Maqu_0168* (102.0 mg/L to 110.2 mg/L) expressing *OPI1* mutant strains.

In addition, isoamyl alcohol concentration was found to be boosted more than 35 % for both BY4742-IB *opi1Δ* (87.2 mg/L) and BY4742-IB (90.1 mg/L) compared to BY4742 strain (64.3 mg/L) (Table 1). Indeed, the expression of the isobutanol pathway enzymes can also boost isoamyl alcohol levels, also as a result of overlapping synthesis pathway with the isobutanol producing pathway.

### High-cell-density fermentation for FASBEs production

**Fig. 6:**
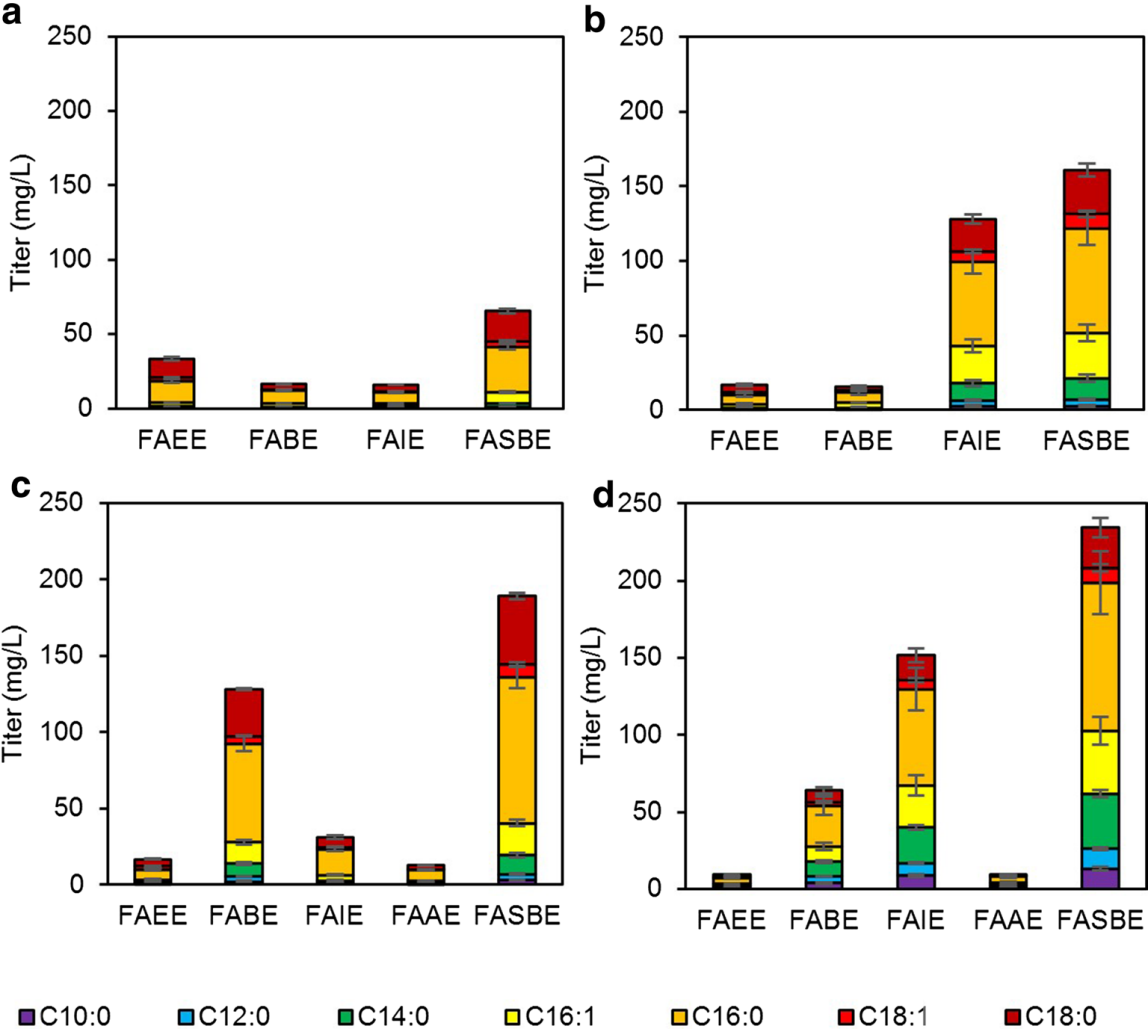
High-cell-density fermentation for FASBEs production of different fatty acid chain lengths. Four strains were chosen for the fermentation: **a** BY4741 *opi1Δ* expressing *ws2*, **b** BY4742 expressing *Maqu_0168*, **c** BY4742-IB *opi1Δ* expressing *ws2* and **d** BY4742-IB *opi1Δ* expressing *Maqu_0168*. All strains were cultured in minimal medium lacking appropriate amino acid and/or nucleotide with 2 % galactose at an initial OD_600_ of ~9. Values are the mean of biological triplicates ±SD at 48 h

We next characterized four best strains for production of FASBEs using high-cell-density fermentation. High-cell-density fermentation would allow rapid and more efficient conversion of sugars into desired products [15]. Cells were inoculated with initial OD_600_ of 9 (Additional file 1: Figure S6). Figure 6 and Additional file 1: Figure S7 shows that yeast strain BY4742 *opi1Δ* expressing *ws2* produced 33.4 mg/L FAEEs, 16.2 mg/L FABEs and 15.9 mg/L FAIEs, giving a total of 65.5 mg/L FASBEs (15.9 mg/gDCW). Yeast strain BY4742 expressing *Maqu_0168* produced 16.9 mg/L FAEEs, 15.8 mg/L FABEs and 128.1 mg/L FAIEs, giving a total of 160.8 mg/L FASBEs (30.2 mg/gDCW). Yeast strain BY4742-IB *opi1Δ* expressing *ws2* produced 16.9 mg/L FAEEs, 127.9 mg/L FABEs, 31.4 mg/L FAIEs and 12.9 mg/L FAAEs, giving a total of 189.1 mg/L FASBEs (42.2 mg/gDCW). Yeast strain BY4742-IB *opi1Δ* expressing *Maqu_0168* produced 9.43 mg/L FAEEs, 63.8 mg/L FABEs, 151.5 mg/L FAIEs and 9.48 mg/L FAAEs, giving a total of 234.2 mg/L FASBEs (50.9 mg/gDCW). The majority of FASBEs had fatty acid components of carbon chain lengths of C16 and C18, while esters with shorter chain length fatty acids were minor products. Strains expressing *ws2* gave a higher proportion of longer chain FASBEs (fatty acid components of carbon chain lengths of C16 and C18, ~92 %), whereas for strains expressing *Maqu_0168*, the longer chain FASBEs proportion was ~80 %. In addition, strains expressing *ws2* resulted in a product profile that had higher proportion of FASBEs with saturated fatty acid components (~84 %) compared to strains expressing *Maqu_0168* which gave ~77 % FASBEs with saturated fatty acid components. All FASBEs with unsaturated fatty acid components were monounsaturated.

## Conclusions

Concerns about limited supply of nonrenewable fossil fuels and the environmental impact of their usage continue to encourage discovery and development of renewable advanced biofuels, such as alcohols, alkanes, fatty acid esters and isoprenoids [19–23]. Recently, Liu and coworkers engineered *Escherichia coli* to produce fatty acid esters with short- and branched-chain alcohol moieties from glycerol [22, 23]. Introduction of 2-keto acid pathway and metabolic engineering of the fatty acid pathway together with expression of a wax ester synthase enzyme from *Acinetobacter baylyi* enabled production of a range of fatty acid esters, including ethyl, propyl, isobutyl, butyl and isoamyl esters [22]. Further introduction of branched fatty acid biosynthetic pathway resulted in branched fatty acid branched-chain esters [23].

Here, we have engineered yeast *S. cerevisiae* to produce and accumulate FASBEs that can be used as biodiesel, including FAEEs, FABEs, FAIEs and FAAEs. However, challenges remain before large-scale bioproduction can be considered. First, even though we have produced up to 230 mg/L FASBEs, which is the highest reported fatty acid ester production titer in yeast to date, further manipulation of the cells through increase of intermediate supply and use of gene regulatory tools are required to maximize the production potential of yeast [24–27]. Second, the products were found to be accumulated intracellularly. Strategies for product recovery need to be developed to enable cell reuse [28, 29].

In this work, it was found that wax synthases ws2 and Maqu_0168 resulted in different product profiles where the former preferentially produced FAEEs, FABEs and FAAEs and the latter produced mostly FAIEs. In addition, more FABEs and FAIEs were produced compared to FAEEs, even though ethanol levels were much higher than the other alcohols (Table 1). This can be attributed to higher specific activity of wax synthases for longer chain alcohols [5, 18]. Hence, the identification and use of wax synthases according to substrate preference and desired products are crucial for improved biodiesel production.

In summary, we engineered yeast to produce FASBEs using endogenously synthesized fatty acids and alcohols. Two wax ester synthase genes (*ws2 and Maqu_0168*) were found to catalyze the formation of FASBEs, with different alcohol preferences. To boost the ability of yeast to produce the FASBEs, gene deletions (*OPI1* and *RPD3*) were carried out to increase flux towards fatty acyl-CoAs. In addition, isobutanol pathway enzymes (Ilv2, Ilv5, Ilv3, Aro10 and Adh7) targeted into the mitochondria were overexpressed, producing branched alcohols for esterification. By combining these engineering strategies, and through high-cell-density fermentation, over 230 mg/L FASBEs were produced, which is the highest titer reported in yeast to date.

## Methods

### Yeast strains and media

The yeast strains used in this study were derived from *S. cerevisiae* strain BY4742 (*MATα; his3Δ1; leu2Δ0; lys2Δ0; ura3Δ0*) (ATCC, Manassas, VA, USA) (Table 2). Isobutanol producing strain M12 derived from BY4742 was a gift from Dr. Jifeng Yuan (National University of Singapore). Plasmids used are listed in Table 3. DNA primers used for PCR are listed in Additional file 1: Table S1. *E. coli* strain Top10 was used for transformation and amplification of plasmids, grown at 37 °C in Luria–Bertani broth supplemented with 100 μg/mL ampicillin. Yeast extract and peptone were obtained from BD (Franklin Lakes, NJ, USA), whereas other chemicals were purchased from Sigma Aldrich (St. Louis, MO, USA), unless otherwise stated. Yeast strains were grown in minimal medium (yeast nitrogen base 6.7 g/L, yeast synthetic drop-out medium supplements—Ura- 1.92 g/L, d-glucose 20 g/L) at 30 °C with 225 rpm shaking.

**Table 2:** Strains used in this study

Strains	Genotype	Source
BY4742	*MATα his3Δ1 leu2Δ0 lys2Δ0 ura3Δ0*	EUROSCARF
BY4742-IB	BY4742 with isobutanol pathway integrated	Yuan and Ching [17]
pESC BY4742	BY4742 (pESC-URA)	This work
pESC BY4742-IB	BY4742-IB (pESC-URA)	This work
ws2 BY4742	BY4742 (pESC-GAL10p-ws2)	This work
Maqu_0168 BY4742	BY4742 (pESC-GAL10p-Maqu_0168)	This work
ws2 BY4742-IB	BY4742-IB (pESC-GAL10p-ws2)	This work
Maqu_0168 BY4742-IB	BY4742-IB (pESC-GAL10p-Maqu_0168)	This work
BY4742 *rpd3Δ*	*rpd3Δ*::kanMX derived from BY4742	This work
BY4742 *opi1Δ*	*opi1Δ*::kanMX derived from BY4742	This work
BY4742-IB *opi1Δ*	*opi1Δ*::kanMX derived from BY4742-IB	This work
ws2 BY4742 *rpd3Δ*	BY4742 *rpd3Δ* (pESC-GAL10p-ws2)	This work
Maqu_0168 BY4742 *rpd3Δ*	BY4742 *rpd3Δ* (pESC-GAL10p-Maqu_0168)	This work
ws2 BY4742 *opi1Δ*	BY4742 *opi1Δ* (pESC-GAL10p-ws2)	This work
Maqu_0168 BY4742 *opi1Δ*	BY4742 *opi1Δ* (pESC-GAL10p-Maqu_0168)	This work
ws2 BY4742-IB *opi1Δ*	BY4742-IB *opi1Δ* (pESC-GAL10p-ws2)	This work
Maqu_0168 BY4742-IB *opi1Δ*	BY4742-IB *opi1Δ* (pESC-GAL10p-Maqu_0168)	This work

**Table 3:** Plasmids used in this study

Plasmids	Description	Source
pUG6	Plasmid containing loxP-kanMX-loxP deletion cassette	EUROSCARF
pESC-URA	2μ plasmid (URA marker)	Agilent
pESC-GAL10p-ws2	pESC-URA with *ws2* cloned in	This work
pESC-GAL10p-Maqu_0168	pESC-URA with *Maqu_0168* cloned in	This work

### Cloning procedures

Restriction enzymes used were procured from New England Biolabs (Ipswich, MA, USA) and digestions were conducted according to the recommended protocols. PCR amplifications were performed with iProof High Fidelity DNA Polymerase (Bio-Rad, Hercules, CA, USA) at suggested conditions. Ligations were carried out with T4 DNA Ligase (New England Biolabs) at 16 °C. Gel extractions were performed using QIAquick gel extraction kit (Qiagen, Venlo, The Netherlands). Minipreps of plasmids from *E.coli* were carried out using QIAprep Spin Miniprep kit. Standard chemical transformation methods for *E.coli* were applied for transformation of ligation mixtures. The LiAc/PEG technique was used for transformation of yeast cells.

### Plasmids and yeast knockout strains construction

To construct plasmids expressing wax ester synthase proteins, genes *ws2* and *Maqu_0168* were codon optimized and synthesized using GeneArt (Life Technologies, Carlsbad, CA, USA) (Additional file 1: Table S2). PCR was carried out using primers ws-f-EcoRI and ws-r-NotI for *ws2* and Maqu-f-EcoRI and Maqu-r-NotI for *Maqu_0168*. The PCR products were then gel extracted and digested using EcoRI and NotI, and ligated into pESC-URA, also digested with the same enzymes. Correct clones were confirmed by colony PCR and plasmid sequencing.

To create yeast knockout strains, gene deletion cassettes were amplified using PCR with primers as shown in Additional file 1: Table S1 and pUG6 as the template. The deletion cassettes were then transformed into yeast strains and screened on YPD plates containing 200 μg/mL G418 and verified by PCR.

Growth parameters of engineered strains including growth rate and yield of biomass are shown in Additional file 1: Table S3. Dry cell weight (DCW) was calculated from the optical density at 600 nm (1 OD_600_ = 0.262 gDCW/L).

### Determining FASBEs production

For quantifying FASBEs produced in different engineered yeast strains, single colonies were pre-cultured overnight in 3 mL minimal medium with glucose as carbon source. The cells were then inoculated into 50 mL fresh minimal medium with 1.8 % galactose and 0.2 % glucose in 250 mL glass flasks at an initial OD_600_ of 0.05. At the stated time points, fatty acid esters were extracted and quantified as follows. Cells (2.5 mL) were centrifuged and the supernatant decanted, and 0.5 mL of autoclaved water was added to re-suspend the cells. Next, the cells were transferred to a 2 mL Fastprep tube (MP Biomedicals, Santa Ana, CA, USA) added with 0.3 g glass beads (425–600 μm). The cells were lyzed using a Fastprep-24 homogenizer, at 6 m/s for 30 s, put on ice for 2 min and repeated for 10 times. Hexane (0.5 mL) spiked with methyl heptadecanoate as internal standard was then added to the lysed cells, followed by vigorous vortex to extract the fatty acid esters. The mixture was centrifuged at maximum speed to separate aqueous and organic layers, where 200 μL of product containing hexane was transferred to GC vials for quantification. GC/MS (7890B GC system, 5977A MSD, Agilent Technologies, Santa Clara, CA, USA) analysis was carried out with a HP-5 ms column (Agilent Technologies) with a 0.25 μm film thickness, 0.25 mm diameter, and 30 m length. The GC program was set as follows: an initial temperature of 45 °C was maintained for 1.5 min, followed by ramping to 180 °C at a rate of 15 °C/min, where it was held for 3 min. The temperature was then ramped to 280 °C at a rate of 10 °C/min, where the temperature was held for 5 min.

### Determining alcohol levels and phospholipid content

To quantify alcohols produced by the engineered yeast strains, single colonies were pre-cultured overnight in 1 mL minimal medium with glucose as carbon source. The cells were then inoculated into 5 mL fresh minimal medium with 1.8 % galactose and 0.2 % glucose in 50 mL tubes at an initial OD_600_ of 0.05. After 48 h growth, alcohols were extracted as follows. Cell culture (0.5 mL) was transferred to a clean 1.5 mL tube, and 0.5 mL hexane spiked with 1-butanol as internal standard was then added to the cells, followed by vigorous vortex to extract the alcohols. The mixture was then centrifuged at maximum speed to separate aqueous and organic layers, where 300 μL of alcohol containing hexane was transferred to GC vials for quantification. GC/MS analysis was carried out with a DB-FFAP column (Agilent Technologies) with a 0.25 μm film thickness, 0.25 mm diameter, and 30 m length. The GC program was set as follows: an initial temperature of 45 °C was maintained for 15 min, followed by ramping to 240 °C at a rate of 30 °C/min, where it was held for 3 min. To analyze the phospholipid concentration, yeast cells were centrifuged and washed with autoclaved deionized water, followed using a phospholipid assay kit (Abnova, Taipei, Taiwan) to determine the concentration of phospholipids according to the manufacturer’s instructions.

### High-cell-density fermentation for FASBEs production

To measure the production of FASBEs with fermentation at high cell density, single colonies of the engineered yeast strains were pre-cultured overnight in 3 mL minimal medium with glucose as carbon source. The cells were then rediluted with 50 mL minimal medium with 2 % glucose in 250 mL glass flasks at an initial OD_600_ of 0.25 and grown for a further 24 h. Next, the cells were centrifuged and inoculated into 50 mL fresh minimal media with 2 % galactose in 250 mL glass flasks (giving an initial OD_600_ of ~9). After 48 h culture, 2.5 mL of each sample was centrifuged and the FASBEs extracted and quantified as described for FASBEs above.

## Additional file

10.1186/s13068-015-0361-5 Supplementary tables and figures. **Table S1.** List of primers used in this study. **Table S2. **Codon optimized sequence of wax ester synthase genes used in this study. **Table S3.** Growth parameters of engineered yeast strains. **Figure S1.** Relative FAEEs, FABEs or FAIEs production with exogenous alcohol feeding. **Figure S2.** Production of fatty acid esters in engineered yeast expressing ws2. **Figure S3.** Relative phospholipid concentration of engineered yeast strains. **Figure S4.** Production of fatty acid esters in engineered yeast expressing Maqu_0168. **Figure S5.** Growth curves for engineered cells. **Figure S6.** OD_600_ for high cell density fermentation. **Figure S7.** FASBEs production yield corresponding to Fig. 6.

## Abbreviations

FASBE: fatty acid short- and branched-chain alkyl ester
FAME: fatty acid methyl ester
FAEE: fatty acid ethyl ester
FABE: fatty acid isobutyl ester
FAIE: fatty acid isoamyl ester
FAAE: fatty acid active amyl ester
GC/MS: gas chromatography mass spectrometry
OD_600_: optical density at 600 nm
