# Supplementary material for: Metabolic engineering of Saccharomyces cerevisiae for production of fatty acid short- and branched-chain alkyl esters biodiesel
Source: Biotechnol Biofuels. 2015 Nov 4;8:177. doi: 10.1186/s13068-015-0361-5 (PMC4634726; doi:10.1186/s13068-015-0361-5)
Supplement: Supplementary file 1 — 10.1186/s13068-015-0361-5 Supplementary tables and figures. Table S1. List of primers used in this study. Table S2. Codon optimized sequence of wax ester synthase genes used in this study. Table S3. Growth parameters of engineered yeast strains. Figure S1. Relative FAEEs, FABEs or FAIEs production with exogenous alcohol feeding. Figure S2. Production of fatty acid esters in engineered yeast expressing ws2. Figure S3. Relative phospholipid concentration of engineered yeast strains. Figure S4. Production of fatty acid esters in engineered yeast expressing Maqu_0168. Figure S5. Growth curves for engineered cells. Figure S6. OD600 for high cell density fermentation. Figure S7. FASBEs production yield corresponding to Fig. 6. [file 13068_2015_361_MOESM1_ESM.pdf]

Additional File 1

Metabolic engineering of *Saccharomyces cerevisiae* for production of fatty acid short- and branched-chain alkyl esters biodiesel

*Wei Suong Teo, Hua Ling, Ai-Qun Yu, Matthew Wook Chang**

Table S1: List of primers used in this study.

DNA Primer	Sequence (5' to 3')
Maqu-f-EcoR1	AAGAGGAATTCATGACTCCATTGAACCCAACC
Maqu-r-Not1	CTCTTGCGGCCGCTCAGTGATGG
ws-f-EcoR1	AAGAGGAATTCATGAAGAGATTGGGTACTTTGG
ws-r-Not1	CTCTTGCGGCCGCTTAGTGATGG
OPI1 KO-f	TACAGTGCTGATTAAAGCGTGTGTATCAGGACAGTGTTTTTAACGAAGATACTAGTCATTG ATGTCTGAAAATCAACGTTTAGGATTATCAGAGGAAGAGGCAGCTGAAGCTTCGTACGC
OPI1 KO-r	TATTATTCCGTATAATATTATTACTGGTGGTAATGCATGAAAGACCTCAATCTGTCTCGGTT AGTCCTTGCTATCCACGTTGTCCTGAGAGGGCTTTAGCATAGGCCACTAGTGGATCTG
RPD3 KO-f	ATGGTATATGAAGCAACACCTTTTGATCCGATCACGGTCACAGCTGAAGCTTCGTACGC
RPD3 KO-r	TCAATAGAATTCATTGTCATGCTCAACATGTAGGTCCCGCATAGGCCACTAGTGGATCTG
Kan-r	GCGTTTCCCTGCTCGCAGGT
OPI1 up-f	GCCAAGAAAGCATATCAGGCCAGAA
OPI1 mid-r	GAACTCATCGTCGTTGTAGTCGTAATCATC
RPD3 up-f	CGAAAGGGAAAAACAGAAAAGATACTAGTAGTTG
RPD3 mid-r	CATCAGTATGGAACTGACACATTTCTTGTTTCG

Table S2: Codon optimized sequence of wax ester synthase genes used in this study.

Gene	Sequence (5’ to 3’)
ws2	ATGAAGAGATTGGGTACTTTGGATGCTTCTTGGTTGGCTGTTGAATCTGAAGATACTCCAAT<br>GCATGTTGGTACTTTACAAATCTTCTCATTGCCAGAAGGTGCTCCAGAAACTTTTTTGAGAG<br>ATATGGTTACCAGAATGAAGGAAGCTGGTGATGTTGCTCCACCTTGGGGTTACAAATTGGC<br>TTGGTCTGGTTTTTTGGGTAGAGTTATTGCTCCAGCATGGAAGGTTGATAAGGATATTGATT<br>TGGACTACCACGTTAGACATTCTGCTTTGCCAAGACCAGGTGGTGAAAGAGAATTAGGTAT<br>TTTGGTTTCCAGATTGCACTCCAACCCATTGGATTTTTCAAGACCATTGTGGGAATGCCATG<br>TTATCGAAGGTTTGGAAAACAATAGATTCGCCTTGTACACCAAGATGCATCACTCTATGATT<br>GATGGTATCTCCGGTGTTAGATTGATGCAAAGAGTTTTGACTACCGATCCAGAAAGATGTAA<br>TATGCCACCACCTTGGACTGTTAGACCACATCAAAGAAGAGGTGCTAAGACTGACAAAGAA<br>GCCTCTGTTCCAGCTGCTGTTTCTCAAGCTATGGATGCTTTGAAATTGCAAGCTGATATGGC<br>TCCAAGATTGTGGCAAGCTGGTAATAGATTGGTTCATTCTGTTAGACATCCAGAAGATGGTT<br>TGACTGCTCCTTTTACTGGTCCAGTTTCTGTTTTGAACCATAGAGTTACAGCCCAAAGAAGA<br>TTCGCTACTCAACACTACCAATTAGACAGATTGAAGAACTTGGCTCATGCTTCTGGTGGTTC<br>TTTGAATGATATTGTCTTGTACTTGTGCGGTACTGCCTTAAGAAGATTTTTGGCTGAACAAAA<br>CAACTTGCCAGATACACCATTGACTGCTGGTATTCCAGTTAACATTAGACCAGCTGATGATG<br>AAGGTACTGGTACTCAAATCTCCTTTATGATTGCTTCTTTGGCTACCGATGAAGCTGATCCA<br>TTGAATAGATTGCAACAAATCAAGACCTCTACCAGAAGAGCTAAAGAACACTTGCAAAAGTT<br>GCCAAAGTCTGCTTTGACTCAATACACCATGTTGTTGATGTCCCCATATATCTTACAATTAAT<br>GTCCGGTTTGGGTGGTAGAATGAGACCAGTTTTTAACGTTACCATCTCTAATGTTCCAGGTC<br>CTGAAGGTACATTGTATTATGAAGGTGCTAGATTGGAAGCCATGTACCCAGTTTCTTTGATT<br>GCTCATGGTGGTGCCTTGAACATTACTTGTTTGTCTTATGCTGGTTCCTTGAACTTTGGTTT<br>CACTGGTTGTAGAGATACCTTGCCATCTATGCAAAAATTGGCTGTTTACACTGGTGAAGCCT<br>TGGATGAATTGGAATCTTTGATTTTGCCACCTAAGAAGAGAGCCAGAACTAGAAAACATCAT<br>CATCACCATCACTAA
Maqu_0168	*A*TGACTCCATTGAACCCAACCGACCAATTATTCTTGTGGTTGGAAAAAAGACAACAACCTAT<br>GCACGTTGGTGGTTTACAATTATTCAGTTTTCCTGAAGGTGCCCCAGATGATTATGTTGCTC<br>AATTAGCTGACCAATTAAGACAAAAGACTGAAGTTACCGCCCCATTCAATCAAAGATTGTCT<br>TATAGATTGGGTCAACCAGTTTGGGTTGAAGATGAACATTTGGATTTGGAACACCACTTTAG<br>ATTTGAAGCCTTGCCAACTCCAGGTAGAATCAGAGAATTATTGTCTTTCGTTTCCGCCGAAC<br>ATTCCCATTTGATGGATAGAGAAAGACCAATGTGGGAAGTTCATTTGATCGAAGGTTTGAAG<br>GATAGACAATTTGCCTTGTACACCAAGGTTCACCATTCTTTGGTTGATGGTGTTTCTGCTAT<br>GAGAATGGCTACTAGAATGTTGTCTGAAAACCCTGATGAACATGGTATGCCACCAATTTGG<br>GATTTGCCATGTTTGTCAAGAGATAGAGGTGAATCTGATGGTCATTCTTTGTGGAGATCAGT<br>TACTCATTTGTTGGGTTTGTCCGGTAGACAATTAGGTACTATTCCAACCGTTGCCAAAGAAT<br>TATTGAAAACCATCAATCAAGCCAGAAAGGACCCAGCTTACGATTCTATTTTTCATGCTCCA<br>AGATGCATGTTGAATCAAAAGATTACCGGTTCCAGAAGATTCGCTGCTCAATCTTGGTGTTT<br>GAAGAGAATTAGAGCTGTTTGTGAAGCCTACGGTACTACTGTTAATGATGTTGTTACAGCTA<br>TGTGTGCTGCTGCTTTGAGAACTTACTTGATGAATCAAGATGCCTTGCCAGAAAAACCATTG<br>GTTGCTTTTGTTCCAGTCTCCTTGAGAAGAGATGATTCTTCTGGTGGTAATCAAGTCGGTGT<br>TATTTTGGCTTCATTGCACACTGATGTTCAAGAAGCTGGTGAAAGATTATTGAAGATTCACC<br>ACGGTATGGAAGAAGCCAAACAAAGATATAGACACATGTCCCCAGAAGAAATCGTTAACTAT<br>ACTGCTTTGACTTTGGCTCCAGCTGCTTTCCATTTGTTGACTGGTTTAGCTCCAAAGTGGCA<br>AACTTTCAACGTTGTTATTTCTAACGTTCCAGGTCCATCCAGACCATTATATTGGAATGGTG<br>CTAAATTGGAAGGTATGTACCCAGTTTCCATCGATATGGATAGATTGGCTTTGAACATGACC<br>TTGACCTCTTACAACGATCAAGTCGAATTTGGTTTGATCGGTTGTAGAAGAACTTTGCCATC<br>ATTGCAAAGAATGTTGGACTACTTGGAACAAGGTTTGGCTGAATTGGAATTGAACGCTGGTT<br>TACATCATCATCACCATCACTGA

Table S3: Growth parameters of engineered yeast strains.

**Strain**	**μ_{max}(h^{-1})**	**Y_{sx} (g/g)**
ws2 BY4742	0.337 ± 0.029	0.137 ± 0.002
ws2 BY4742 *opi1Δ*	0.380 ± 0.006	0.129 ± 0.002
ws2 BY4742 *rpd3Δ*	0.317 ± 0.023	0.115 ± 0.002
Maqu_0168 BY4742	0.321 ± 0.029	0.128 ± 0.002
Maqu_0168 BY4742 *opi1Δ*	0.333 ± 0.016	0.116 ± 0.002
Maqu_0168 BY4742 *rpd3Δ*	0.274 ± 0.011	0.112 ± 0.003
pESC BY4742	0.417 ± 0.017	0.129 ± 0.001
ws2 BY4742-IB	0.513 ± 0.012	0.091 ± 0.001
ws2 BY4742-IB *opi1Δ*	0.396 ± 0.055	0.101 ± 0.003
Maqu_0168 BY4742-IB	0.431 ± 0.006	0.056 ± 0.001
Maqu_0168 BY4742-IB *opi1Δ*	0.404 ± 0.049	0.069 ± 0.002
pESC BY4742-IB	0.424 ± 0.038	0.131 ± 0.002

Y_{sx}: yield of biomass on substrate

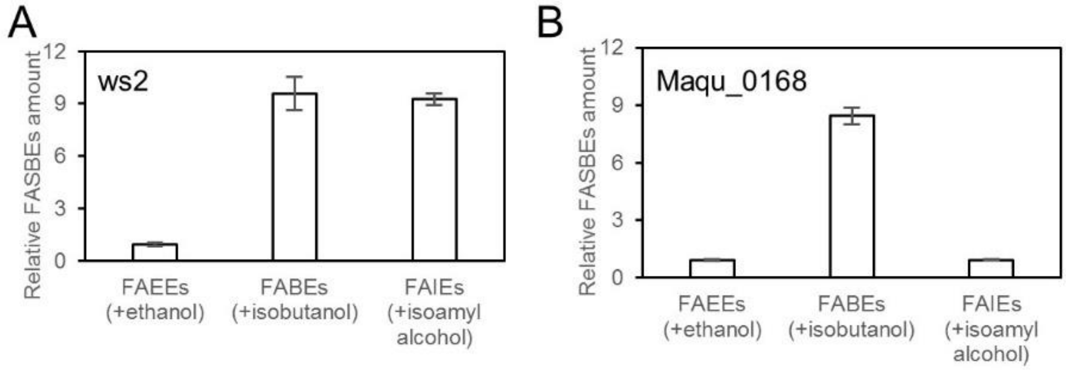

Figure S1: Relative FAEEs, FABEs or FAIEs production with exogenous alcohol feeding. Yeast cells expressing ws2 (A) and Maqu_0168 (B) with exogenous feeding of 0.1% ethanol, isobutanol or isoamyl alcohol were compared with no alcohol feeding cells. Values are the mean of biological duplicates ± SD after 48 h.

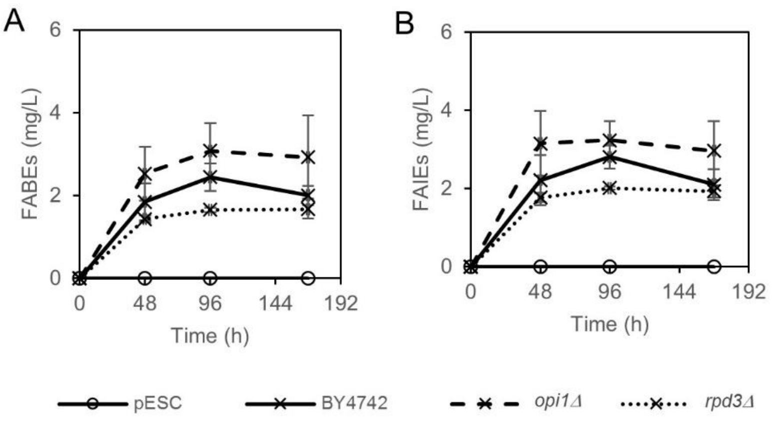

Figure S2: Production of fatty acid esters in engineered yeast expressing ws2. (A) FABEs (B) Total FAIEs. Figures are the same as Figure 3B and C, with adjusted y-axis scales.

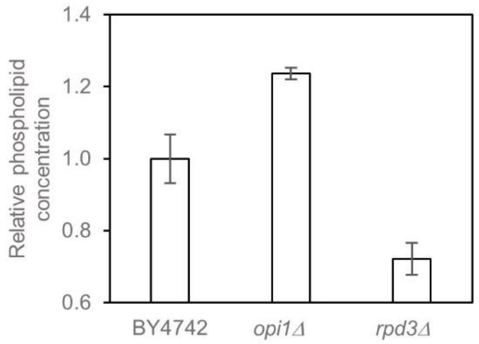

Figure S3: Relative phospholipid concentration of engineered yeast strains. Strains were transformed with empty pESC-URA plasmid and cultured in minimal medium lacking uracil with 0.2% glucose and 1.8% galactose. Absolute phospholipid concentration for BY4742 was 16.2 μM. Values are the mean of biological triplicates ± SD after 48 h.

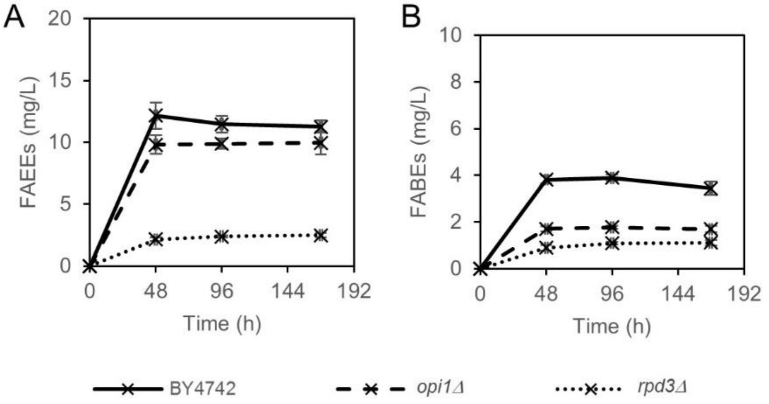

Figure S4: Production of fatty acid esters in engineered yeast expressing Maqu_0168. (A) FAEEs (B) FABEs. Figures are the same as Figure 4A and B, with adjusted y-axis scales.

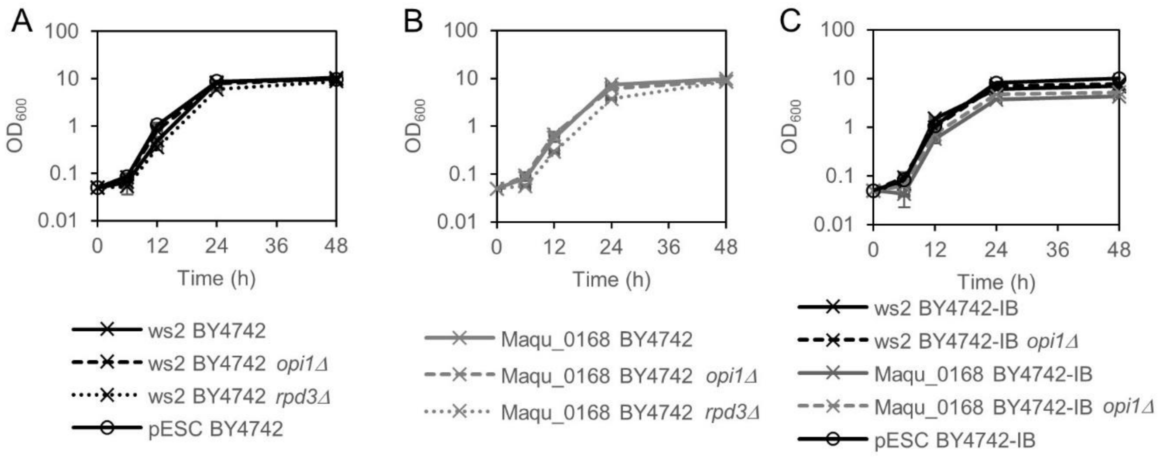

Figure S5: Growth curves for engineered cells. (A) BY4742 and mutants expressing ws2 (B) BY4742 and mutants expressing Maqu_0168 (C) BY4742-IB and OPI1 mutant expressing ws2 or Maqu_0168. Values are the mean of biological triplicates ± SD at 0,6,12,24 and 48 h.

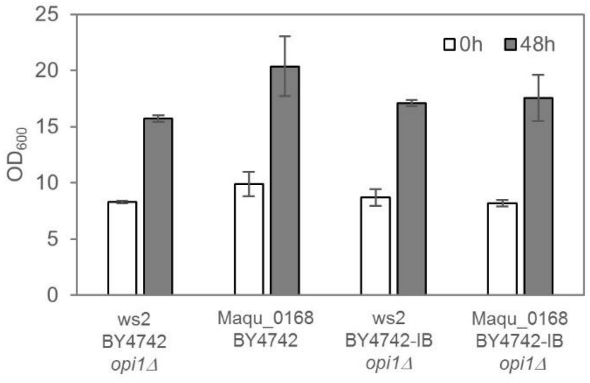

Figure S6: OD_{600} for high cell density fermentation as shown in Figure 6. Values are the mean of biological triplicates ± SD at 48 h.

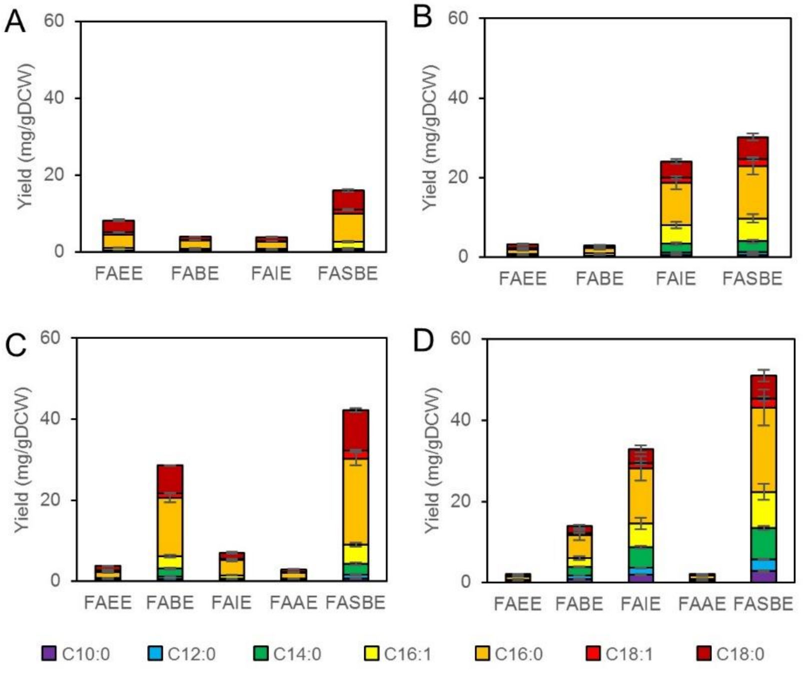

Figure S7: FASBEs production yield corresponding to Figure 6. (A) BY4741 *opi1Δ* expressing *ws2*, (B) BY4742 expressing *Maqu_0168*, (C) BY4742-IB *opi1Δ* expressing *ws2* and (D) BY4742-IB *opi1Δ* expressing *Maqu_0168*.
